# Dose-Response Effect of a Digital Health Intervention During Cardiac Rehabilitation: Subanalysis of Randomized Controlled Trial

**DOI:** 10.2196/13055

**Published:** 2020-02-26

**Authors:** R Jay Widmer, Conor Senecal, Thomas G Allison, Francisco Lopez-Jimenez, Lilach O Lerman, Amir Lerman

**Affiliations:** 1 Department of Health Sciences Research Mayo Clinic Rochester, MN United States; 2 Division of Nephrology and Hypertension Mayo Clinic Rochester, MN United States

**Keywords:** cardiovascular prevention, secondary prevention, online, digital health interventions

## Abstract

**Background:**

Previous data have validated the benefit of digital health interventions (DHIs) on weight loss in patients following acute coronary syndrome entering cardiac rehabilitation (CR).

**Objective:**

The primary purpose of this study was to test the hypothesis that increased DHI use, as measured by individual log-ins, is associated with improved weight loss. Secondary analyses evaluated the association between log-ins and activity within the platform and exercise, dietary, and medication adherence.

**Methods:**

We obtained DHI data including active days, total log-ins, tasks completed, educational modules reviewed, medication adherence, and nonmonetary incentive points earned in patients undergoing standard CR following acute coronary syndrome. Linear regression followed by multivariable models were used to evaluate associations between DHI log-ins and weight loss or dietary adherence.

**Results:**

Participants (n=61) were 79% male (48/61) with mean age of 61.0 (SD 9.7) years. We found a significant positive association of total log-ins during CR with weight loss (*r*^2^=.10, *P*=.03). Educational modules viewed (*r*^2^=.11, *P*=.009) and tasks completed (*r*^2^=.10, *P*=.01) were positively significantly associated with weight loss, yet total log-ins were not significantly associated with differences in dietary adherence (*r*^2^=.05, *P*=.12) or improvements in minutes of exercise per week (*r*^2^=.03, *P*=.36).

**Conclusions:**

These data extend our previous findings and demonstrate increased DHI log-ins portend improved weight loss in patients undergoing CR after acute coronary syndrome. DHI adherence can potentially be monitored and used as a tool to selectively encourage patients to adhere to secondary prevention lifestyle modifications.

**Trial Registration:**

ClinicalTrials.gov (NCT01883050); https://clinicaltrials.gov/ct2/show/NCT01883050

## Introduction

Cardiovascular disease (CVD) is the primary cause for morbidity, mortality, and rising health care–associated costs in the United States [1], with 90% of CVD morbidity and mortality due to preventable risk factors such as poor diet, smoking, and lack of physical activity [2]. Cardiac rehabilitation (CR) is a class IA recommendation by both the American Heart Association and American College of Cardiology after percutaneous coronary intervention (PCI) for acute coronary syndrome [3], and patients with weekly participation in CR following PCI demonstrate a decrease in all-cause mortality [4].

In addition to meta-analytic data demonstrating improved CVD outcomes with digital health intervention (DHI) participation [5], we have demonstrated in a randomized controlled trial (RCT) [6] and a similar-sized feasibility study [7] benefits in intermediate markers of secondary CVD prevention and reductions in rehospitalizations and emergency department visits in patients who are prescribed a DHI for CR. We have also demonstrated a dose-dependent effect of DHI on weight loss and blood pressure in a large cohort of workplace participants seeking primary prevention benefits [8]. It is unclear if weight loss during a DHI RCT occurs in a dose-dependent fashion. Thus, this analysis was designed to evaluate DHI log-in patterns among CR participants assigned to a DHI after PCI and determine if there was an association with weight loss data in these cohorts.

## Methods

### Patient Selection

**Figure 1:**
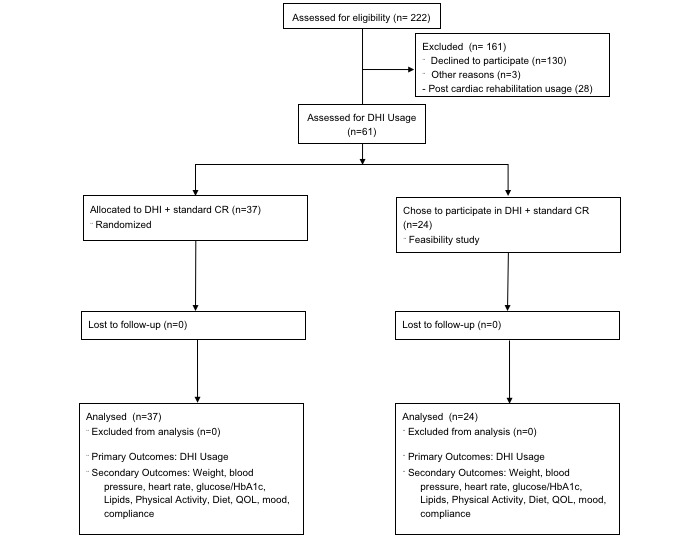
CONSORT diagram for digital health use substudy of initial feasibility study and randomized trial.

Patient data was abstracted from a combination of a feasibility study (n=24) [7] and RCT (n=37) (Figure 1) [6]. Participants were recruited, consented, and enrolled in a prospective fashion after PCI according to an approved Mayo Clinic institutional review board protocol registered at ClinicalTrials.gov [NCT01883050] between August 2013 and February 2015. As described previously [6,7], inclusion criteria included willingness to participate in CR and access to the internet. Reasons for exclusion from the study were primarily due to declining to participate (n=130), other reasons (n=3), and those who had already completed CR (Figure 1). All participants gave written informed consent to participate both in CR and the trial. The study groups consisted of patients entering 3 months of Mayo Clinic CR who agreed to participate in either the feasibility study or RCT. The groups received education on the use of the online and smartphone-based CR program and how to enter their metrics (weight, body mass index, blood pressure, glucose, lipids, diet habits, physical activity, quality of life [QOL], medication adherence, and smoking status) with the help of a study coordinator within 1 week following enrollment.

### Digital Health Intervention

The DHI has been previously described [7,9]. Briefly, the DHI involved reporting dietary and exercise habits and reading educational information on patient healthy lifestyles throughout CR. Those with compatible smartphones (iPhone and Android) were assisted in downloading the appropriate app; those without compatible smartphones used the Web-based portal (not optimized for mobile use). Training consisted of study coordinators instructing the patients on the program use in a 30-minute session during the first week of CR. Patients were prescribed a standard phase II CR program as described previously [4,7] for 36 sessions (approximately 12 weeks). Five patients downloaded the app in the feasibility study, and seven patients downloaded the app in the RCT.

### Data Obtained: Log-Ins and Outcomes

Baseline and 3-month assessments included standard laboratory blood tests for fasting lipid panels and serum glucose values. These data were obtained from the Mayo Clinic cardiovascular health clinic database by a blinded abstractor and underwent statistical review by a blinded statistician. Furthermore, CR staff collected CR data blinded to the group allocation. Most patients in the study group underwent exercise stress testing at baseline and after 3 months per clinical protocol. The patients’ CR providers assessed end points such as blood pressure, height, weight, and the health behavior questionnaires (including diet, physical activity, Dartmouth QOL Index, stress, and smoking status) at baseline and after 3 months in standard fashion. Weight and blood pressure were measured at every CR visit in standard fashion, with weight being assessed with clothes on and shoes off, and blood pressure assessed by BpTRU (BpTRU Medical Devices). Stress scores were answered on a 1 to 10 scale [10], with QOL surveys using the Dartmouth format [11]. Diet scores were calculated by the summation of daily servings of fruits, vegetables, whole grains, and lean proteins with points taken away for daily servings of saturated fats and sweets [7]. Follow-up assessment at 3 months consisted of a replication of the baseline parameters in a similar fashion. All data were confirmed by patient-reported data in the cardiovascular health clinic database; however, only electronic health record data were used for statistical analysis.

Deidentified data were transmitted through Healarium (Healarium Inc) to the investigators for a comprehensive data analysis at the completion of the program. Patient-provided data in the DHI group were collected but not used in the analysis comparing the two groups. Patients who did not initially report for their intake into CR were removed from the analysis as primary and secondary outcomes data could not be assessed and verified.

DHI data were also assembled and transferred in a deidentified manner and included total log-ins, days logged in, educational modules viewed, and tasks completed in total and broken down by subtasks (weight, exercise, blood pressure, glucose, and medications). We also abstracted and analyzed data for nonmonetary-based incentive markers called Healthies, incentive points given to patients after they completed tasks and milestones such as logging in or reaching certain targets for weight, blood pressure, etc. Values for point allotments were prespecified.

### Statistics

Continuous variables were summarized as mean and standard deviation; categorical variables as frequency and percentage. Group comparisons were made using Student *t* tests or Pearson chi-square tests, respectively. Simple linear regressions were used to model associations between total log-ins and total days active versus changes in weight loss, blood pressure, glucose, minutes of exercise per week, food scores, QOL, and stress scores and reported as *r*^2^ and root mean squared error (RMSE) values. Multivariable analysis using linear regression was used to create a model to identify independent predictors of weight loss. All tests are 2-sided with a .05 type I error rate. Analyses were conducted using JM*P* 13.0 (SAS Institute Inc).

## Results

### Baseline Demographics

Baseline demographics revealed similar baseline statistics between both groups (Table 1) demonstrating a predominantly male (48/61, 79%) cohort with a mean age of 61.0 (SD 9.7) years, mean weight of 95.0 (SD 19.7) kg, and mean weight loss of 5.0 (SD 6.5) kg. Median log-ins was 10 (interquartile range 4-37).

**Table 1:** Baseline demographics of participants.

Characteristics		RCT^a^ n=37	Feasibility n=24	*P* value
Age in years, mean (SD)		62.5 (10.7)	60.1 (12.4)	.69
Gender, male, n (%)		30 (81)	18 (75)	.43
**Working status, n (%)**		**—**	**—**	**.67**
	Working	21 (57)	12 (50)	—
	Retired/disabled	16 (43)	10 (42)	—
**Occupation, n (%)**		**—**	**—**	**.68**
	Professional	12 (34)	5 (21)	—
	Skilled labor	13 (37)	11 (46)	—
	Unskilled labor	5 (14)	4 (17)	—
	White collar	5 (14)	4 (17)	—
Married, n (%)		32 (87)	17 (71)	.29
Education in years, mean (SD)		14.7 (2.1)	14.4 (2.1)	.50
Metabolic syndrome, n (%)		16 (44)	8 (33)	.34
Diabetes, n (%)		11 (32)	6 (26)	.73
Hyperlipidemia, n (%)		33 (89)	22 (96)	.79
Hypertension, n (%)		28 (82)	16 (70)	.29
Family history of CVD^a^, n (%)		26 (70)	14 (55)	.65
Current tobacco, n (%)		1 (3)	3 (14)	.24
Weight (kg), mean (SD)		95.8 (19.8)	93.7 (19.8)	.70
Systolic blood pressure (mm Hg), mean (SD)		118.4 (15.9)	124.3 (14.7)	.16
Glucose (mg/dL), mean (SD)		122.3 (45.9)	123.5 (38.5)	.92

^a^RCT: randomized controlled trial.

^b^CVD: cardiovascular disease.

### Log-Ins and Outcomes

**Figure 2:**
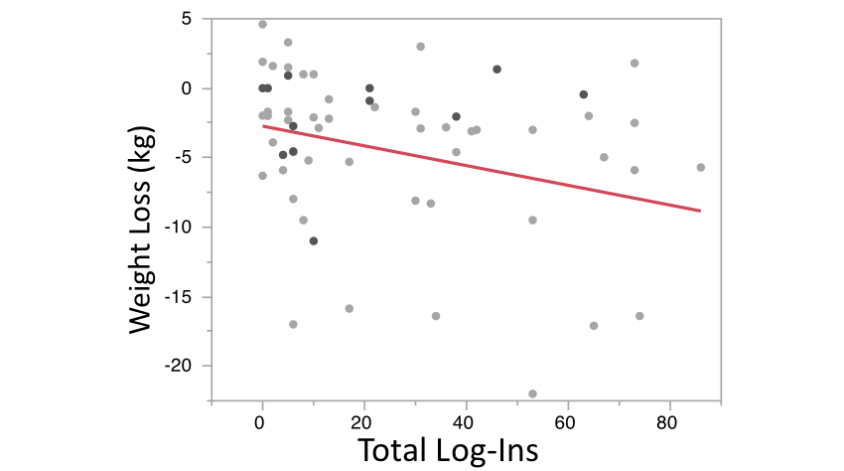
Weight loss (kg) compared with number of log-ins during the 3-month cardiac rehabilitation period (*r*^2^=.10, *P*=.03).

**Figure 3:**
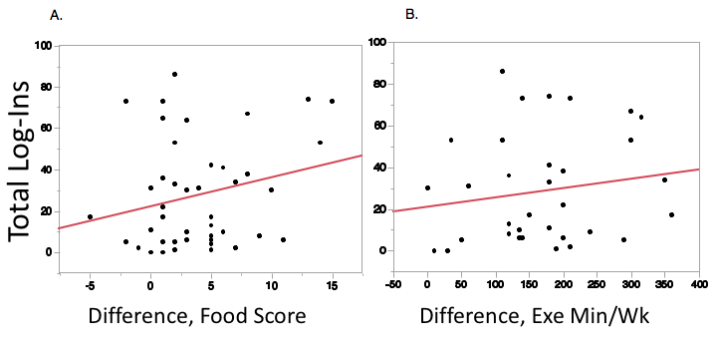
Number of log-ins during 3 months of cardiac rehabilitation compared with (A) diet scores (*r*^2^=.05, *P*=.12) and (B) minutes of weekly exercise (*r*^2^=.03, *P*=.36).

There was a significant association of total log-ins during CR with weight loss (*r*^2^=.10, RMSE=5.69, *P*=.03; Figure 2). This same statistically significant association was not seen when total log-ins were regressed against differences in systolic blood pressure (*r*^2^=.01, RMSE=15.29, *P*=.48), reductions in total cholesterol (*r*^2^=.04, RMSE=51.92, *P*=.16), reductions in low-density lipoprotein cholesterol (*r*^2^=.06, RMSE=46.64, *P*=.11), differences in blood glucose (*r*^2^=.001, RMSE=30.76, *P*=.63), or QOL as assessed by Dartmouth QOL Index (*r*^2^=.07, RMSE=4.47, *P*=.15). Participants who logged their weight more frequently had greater weight loss (*r*^2^=.10, RMSE=5.57 *P*=.01), and those who logged dietary habits at a higher rate had improved dietary adherence (*r*^2^=.42, RMSE=4.07, *P*=.03). Total log-ins were not significantly associated with differences in dietary adherence (*r*^2^=.05, RMSE=4.06, *P*=.12) or improvements in minutes of exercise per week (*r*^2^=.03, RMSE=87.22, *P*=.36; Figure 3). A multivariable model adjusting for age, sex, marital status, and work status showed that total log-ins was still significantly associated with weight loss. This model demonstrated that for every log-in users experienced a 0.08 (SD 0.04) kg weight reduction (*P*=.03).

### Rewards and Outcomes

**Figure 4:**
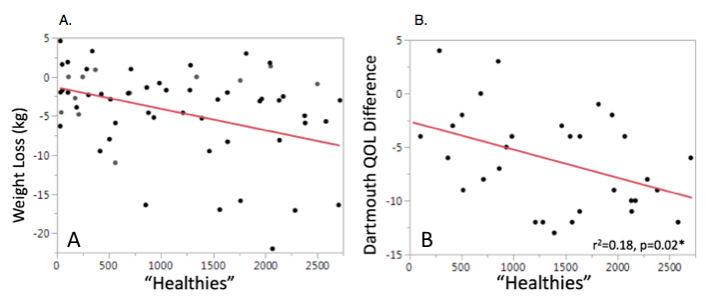
Increased collection of Healthies, nonmonetary point-based incentives, was significantly associated with (A) improved weight loss and (B) improvements in Dartmouth Quality of Life.

Similarly, there was a significant correlation between Healthies and weight loss (*r*^2^=.15, RMSE=5.51, *P*=.006; Figure 4A) and Healthies and improvement in Dartmouth QOL Index (*r*^2^=.18, RMSE=4.19, *P*=.02; Figure 4B). These same significant associations were not seen between Healthies and change in systolic blood pressure (*r*^2^=.03, RMSE=15.15, *P*=.24), reductions in total cholesterol (*r*^2^=.05, RMSE=51.69, *P*=.13), reductions in low-density lipoprotein cholesterol (*r*^2^=.05, RMSE=46.7, *P*=.12), differences in blood glucose (*r*^2^=.002, RMSE=30.84, *P*=.79), or improvements in food scores (*r*^2^=.06, RMSE=4.04, *P*=.12). A multivariable model adjusting for age, sex, marital status, and work status showed that Healthies were also associated with weight loss, and that for every point users earned there was a concomitant 0.003 (SD 0.001) kg weight reduction (*P*=.008).

### Intraprogram Items and Outcomes

**Figure 5:**
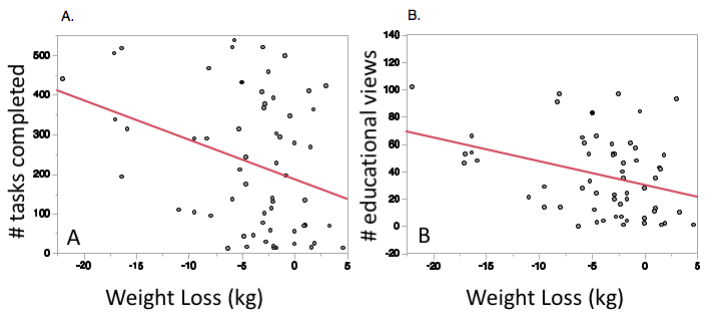
Increased weight loss was associated with (A) an increased number of digital health tasks completed (*r*^2^=.10, *P*=.01) and (B) the number of passive educational views (*r*^2^=.11, *P*=.009) during 3 months of standard cardiac rehabilitation.

Educational modules viewed (*r*^2^=.11, RMSE=5.55, *P*=.009) and number of tasks completed (*r*^2^=.10, RMSE=5.50, *P*=.01) were significantly associated with weight loss (Figure 5). There was also a significant association between number of modules viewed and increase in minutes of exercise per week (*r*^2^=.19, RMSE=80.04, *P*=.01) and between improvements in QOL and number of tasks completed (*r*^2^=.13, RMSE=4.31, *P*=.04). There were no statistically significant associations among number of modules viewed or tasks completed and changes in blood pressure, cholesterol, or glucose.

## Discussion

### Principal Findings

In this study, we have demonstrated that there is a significant association between total log-ins and weight loss in patients in CR assigned to a DHI. These data support the notion of a dose-dependent effect of a DHI on weight loss and extend our previous work highlighting the success of a DHI in improving weight loss for both primary and secondary prevention [6-8]. Interestingly, increased log-ins were not associated with improved dietary adherence or increased exercise frequency; however, patients who logged weight and dietary information had a significant association with improved weight loss and dietary habits. We also demonstrate that nonmonetary incentive points, labeled Healthies, can be an important driver of improved weight loss and QOL. These data related to DHI log-ins and metrics related to secondary CVD prevention could have an important impact on DHI design toward efficacious behavior change.

### Digital Health Intervention Log-Ins and Outcomes

Despite the growing prevalence of digital/mobile health tools in health care with more than 100,000 medically related apps available for download, there are sparse data to show an overall benefit let alone promise of a dose-dependent effect of DHIs. There are data supporting the notions that increased follow-up frequency improves weight loss in a bariatric surgery population [12] and increased telephone contact after discharge can have a positive impact on patient engagement [13]. While these pieces of information are not surprising based on our prior work in primary prevention [8], the dose-dependent effect is one possible explanation for the neutral findings of the South Asian Heart Risk Assessment (SAHARA) trial. This promising and well-executed combination of precision and digital medicine had a positive initial feasibility component [14] that did not carry over to the RCT [15]. In fact, the RCT was entirely neutral showing no difference in the digital arm compared with the control arm in an educated Western population found to be at high risk for CVD based on genetic screening. One notable difference in the two components of the study, aside from randomization, was the reduction in digital contacts from nearly 4 times per week (mean of 2.6 log-ins and at least one weekly reminder) in the feasibility trial to 2 per week in the RCT. There is almost certainly an optimal number of digital touches to maximize the adjunctive effect of digital health on CVD prevention, which also likely varies with each individual modality and patient population. This should be an important lesson in designing future DHIs and carefully considered in future research endeavors.

### Engagement and Weight Loss

Our study is congruent with prior subanalyses showing that improved DHI use equates with improved target attainment and intentions toward behavior change [16,17]. Our study evaluated weight loss on a population in which nearly 40% had metabolic syndrome (Table 1)—clearly a target rich sample in need of weight loss. Clinically speaking, these data demonstrate that for approximately every 10 log-ins, participants lost approximately 1 kg in the absence of increased dietary or exercise adherence (Figures 2 and 3). This is a potentially powerful weight loss intervention in a group with a large majority being overweight and plurality having metabolic syndrome. Interestingly, increased log-ins did not correlate with improved dietary adherence or minutes of exercise performed per week (Figure 3). Moreover, we showed that increased weight loss was seen with both increased number of active tasks performed and passive educational modules performed. So while there might not be a specific component responsible for the weight loss, an interesting piece of data from this study involves tracking the nonmonetary incentives, Healthies. Insurance incentives were an important driver in adherence in our previous population-based work [8,9], and it could be that this portion of the program encouraged adherence and engagement to support a healthy secondary CVD prevention lifestyle. Further work on the importance of incentives and the extent to which these digital programs should be incentivized is another area ripe for further thought and investigation. We are unable to delineate what specific aspect of logging in with increased frequency led to the improvement in weight loss but believe incentives—even if nonmonetary—may play an important role in engagement and potentially increased QOL.

### Limitations

Despite the positive overall message supporting a dose-dependent effect of a DHI on weight loss, there are a few limitations in this study. Notably, this is a willing convenience sample comprising a feasibility/pilot study and the subsequent RCT. However, there were not substantial changes to the protocols among the two sections of the overall project, and the baseline demographics are similar. Another limitation is the lack of standardization on how to quantify use in the digital/mobile community. This has been previously studied in a systematic review which elegantly details that justifications for use are usually lacking in the assessment of DHI adherence [18]. Certainly in this substudy this idea presents a post hoc challenge, and thus we evaluated the most convenient metric, total log-ins, which appeared to match total days active in our statistical analyses. The ability to obtain such multitudinous and granular digital data creates a research dilemma as to what metrics to analyze. This is an understudied area and one that will require clinicians, engineers, informaticists, and behavioralists to carefully study the available data and determine the best way to monitor digital/mobile use. Finally, although we report repeat CVD events in our prior RCT publication, this information was not analyzed with regard to use. First, these small numbers would likely lead to an underpowered sample and no appreciable conclusions. Second, once the patients had a rehospitalization, they likely suspended their CR program and their app use therefore reducing use after the event and giving biased data for the final analysis. Ultimately, hard CVD outcomes and use metrics will need further analysis in a larger, more nuanced randomized trial.

### Conclusion

We are able to demonstrate a use-dependent effect of DHIs on secondary prevention with regard to weight loss in patients participating in standard CR. Adherence metrics should be recorded and reported in mobile/digital health trials, and further work should be done to elucidate the most appropriate use metrics in these trials. Furthermore, these data support the notion that increased contact with patients through mobile/digital mechanisms can have additive benefits in terms of improved body weight profiles.

## Abbreviations

CVD: cardiovascular disease
CR: cardiac rehabilitation
DHI: digital health intervention
PCI: percutaneous coronary intervention
QOL: quality of life
RCT: randomized controlled trial
RMSE: root mean squared error
SAHARA trial: South Asian Heart Risk Assessment
